# High frequency percussive ventilation increases alveolar recruitment in early acute respiratory distress syndrome: an experimental, physiological and CT scan study

**DOI:** 10.1186/s13054-017-1924-6

**Published:** 2018-01-11

**Authors:** Thomas Godet, Matthieu Jabaudon, Raïko Blondonnet, Aymeric Tremblay, Jules Audard, Benjamin Rieu, Bruno Pereira, Jean-Marc Garcier, Emmanuel Futier, Jean-Michel Constantin

**Affiliations:** 1 Departement de Médecine Périopératoire (MPO), Hôpital Estaing, Centre Hospitalier Universitaire (CHU) Clermont-Ferrand, 1 place Lucie Aubrac, Clermont-Ferrand, F-63003 France; 2 Université Clermont Auvergne, Laboratoire Universitaire GReD, UMR/CNRS 6293, INSERM U1103, Clermont-Ferrand, F-63003 France; 3 Département d’Anesthésie et de Réanimation, Centre Hospitalier Universitaire (CHU) Saint-Etienne, Saint-Etienne, F-42000 France; 4 Délégation à la Recherche Clinique et à l’Innovation (DRCI), Centre Hospitalier Universitaire (CHU) Clermont-Ferrand, Clermont-Ferrand, F-63000 France; 5 Département de Radiologie, Centre Hospitalier Universitaire (CHU) Clermont-Ferrand, Clermont-Ferrand, F-63003 France

**Keywords:** High frequency percussive ventilation, Acute respiratory distress syndrome, Alveolar hyperinflation, Lung morphology, Alveolar recruitment, Mechanical ventilation

## Abstract

**Background:**

High frequency percussive ventilation (HFPV) combines diffusive (high frequency mini-bursts) and convective ventilation patterns. Benefits include enhanced oxygenation and hemodynamics, and alveolar recruitment, while providing hypothetic lung-protective ventilation. No study has investigated HFPV-induced changes in lung aeration in patients with early acute respiratory distress syndrome (ARDS).

**Methods:**

Eight patients with early non-focal ARDS were enrolled and five swine with early non-focal ARDS were studied in prospective computed tomography (CT) scan and animal studies, in a university-hospital tertiary ICU and an animal laboratory. Patients were optimized under conventional “open-lung” ventilation. Lung CT was performed using an end-expiratory hold (Conv) to assess lung morphology. HFPV was applied for 1 hour to all patients before new CT scans were performed with end-expiratory (HFPV EE) and end-inspiratory (HFPV EI) holds. Lung volumes were determined after software analysis. At specified time points, blood gases and hemodynamic data were collected. Recruitment was defined as a change in non-aerated lung volumes between Conv, HFPV EE and HFPV EI. The main objective was to verify whether HFPV increases alveolar recruitment without lung hyperinflation. Correlation between pleural, upper airways and HFPV-derived pressures was assessed in an ARDS swine-based model.

**Results:**

One-hour HFPV significantly improved oxygenation and hemodynamics. Lung recruitment significantly rose by 12.0% (8.5–18.0%), *P =* 0.05 (Conv-HFPV EE) and 12.5% (9.3–16.8%), *P* = 0.003 (Conv-HFPV EI). Hyperinflation tended to increase by 2.0% (0.5–2.5%), *P* = 0.89 (Conv-HFPV EE) and 3.0% (2.5–4.0%), *P* = 0.27 (Conv-HFPV EI). HFPV hyperinflation correlated with hyperinflated and normally-aerated lung volumes at baseline: *r* = 0.79, *P* = 0.05 and *r* = 0.79, *P* = 0.05, respectively (Conv-HFPV EE); and only hyperinflated lung volumes at baseline: *r* = 0.88, *P* = 0.01 (Conv-HFPV EI). HFPV CT-determined tidal volumes reached 5.7 (1.1–8.1) mL.kg^-1^ of ideal body weight (IBW). Correlations between pleural and HFPV-monitored pressures were acceptable and end-inspiratory pleural pressures remained below 25cmH_2_0.

**Conclusions:**

HFPV improves alveolar recruitment, gas exchanges and hemodynamics of patients with early non-focal ARDS without relevant hyperinflation. HFPV-derived pressures correlate with corresponding pleural or upper airways pressures.

**Trial registration:**

ClinicalTrials.gov, NCT02510105. Registered on 1 June 2015. The trial was retrospectively registered.

**Electronic supplementary material:**

The online version of this article (doi:10.1186/s13054-017-1924-6) contains supplementary material, which is available to authorized users.

## Background

Conventional mechanical ventilation (CMV) is a cornerstone treatment for acute respiratory distress syndrome (ARDS). However, although being life-saving, CMV can induce ventilator-induced lung injury (VILI) [1]. Open-lung ventilation (OLV) can be applied to improve gas exchange and decrease VILI. Different strategies can be used to perform OLV. Conventional ventilation, combining low tidal volume [2], tailored positive end-expiratory pressure (PEEP) [3], prone positioning [4] and recruitment maneuvers (RM) [5] currently comprise the regular approach. High frequency ventilation, including high frequency oscillatory ventilation (HFO), high frequency percussive ventilation (HFPV) and jet ventilation, have been used. Two recent large randomized controlled trials [6, 7] argued for withdrawal use of HFO in adult patients with ARDS. For technical and safety issues, jet ventilation is no longer used in the ICU. HFPV has been used in neonates [8], burn patients [9–12], trauma patients [13] and patients with chronic obstructive lung disease [14]. HFPV has also been used in ARDS [15], or as rescue therapy [16, 17]. Its theoretical properties include the delivery of low tidal volume ventilation with effective recruitment and enhanced secretion clearance [18].

Gases are administered through pulsatile Flow Ventilation^TM^ Phasitron®, an open circuit device that is believed to adapt ventilation to patient lung volumes, regardless of compliance [19]. During HFPV, high frequency oscillatory diffusive ventilation is superimposed to conventional tidal volume convective ventilation, resulting in a rapid increase in arterial oxygenation [15, 18]. However, despite positive effects on lung alveolar recruitment [18], hyperinflation might be induced by HFPV during ARDS, thus possibly limiting its use only as a rescue therapy in severe ARDS. Therefore, we designed a study to determine changes in lung aeration assessed by CT scan. Moreover, monitoring of alveolar pressures and especially plateau pressure is not obtained from the HFPV Monitron®, since no end-inspiratory hold is possible. We conducted an animal experiment in a pig model of ARDS to monitor upper airway and pleural pressures, in order to investigate correlation between Monitron®-based and pressures recorded *in vivo*. Some of the results of this study have been previously reported in the form of an abstract [20].

## Methods

Additional details are provided in Additional file 1.

### Human studies

#### Ethical statements

Our institutional review board approved the protocol (CPP Sud-Est VI, approval number AU 1138). All participants, or their next-of-kin, provided written consent to participate in this study. The clinical trial is registered at http://www.clinicaltrials.gov (NCT02510105).

#### Study design

Consecutive patients were enrolled in this prospective non-randomized monocentric study within 24 hours of moderate to severe ARDS onset [21]. CMV was optimized by the ICU physician, following the ExPress study settings aimed at increasing alveolar recruitment [3] (Engström Carestation, General Electrics Healthcare). HFPV settings were obtained following manufacturer’s recommendations and equivalent to previously published ones [13, 15]. Further information is in Additional file 1: Table S1, with a pressure waveform example.

#### CT protocol

Baseline lung CT was performed during CMV end-expiratory holds. Lung morphology was assessed following ARDS study group criteria for the CT scan [22]. Diffuse and patchy patterns were considered non-focal [23].

Patients were ventilated with HFPV for 1 hour using the stand-alone ventilator VDR-4 (Volumetric Diffusive Respirator, Percussionaire® Corporation), with a mandatory maximal mean pressure (30 cmH_2_O) and a CMV equivalent mean PEEP. CT was performed during end-inspiratory and end-expiratory holds, by clamping the endotracheal tube. Arterial blood gases were obtained prior to inclusion (time 0 (T0)), every 10 minutes during HFPV (T10–T60) and 10 minutes after resuming CMV (T-after).

#### CT scan analyses

CT scans were computed to obtain 5-mm-thick contiguous sections (Advance Workstation, General Electrics Healthcare). Qualitative assessment of lung aeration, was obtained using the UCLA color-coding table (http://osirixfoundation.com/, OsiriX, Switzerland) and digital post-processing to convert purple into white pixels (http://gimp.org, GNU Image Manipulation Program, Version 2.8.20). Quantitative assessment was performed using dedicated software (Maluna 3.17, University Hospital of Göttingen, Germany). Regions of interest were drawn manually including only lung parenchyma and excluding large vessels and bronchi. Lung gas content, lung weight and aeration distribution were measured as previously reported [23]. Lung tissue aeration was divided into four compartments according to their Hounsfield Units (HU): hyperinflated (densities from −1000 to −900 HU), normally aerated (−900 to −500 HU), poorly aerated (−500 to −100 HU) and non-aerated tissue (−100 to 100 HU). Two ICU physicians (TG and JMC) and a senior radiologist (JMG) reviewed CT scans and manual drawings.

Recruitment was computed during both end-inspiratory and end-expiratory holds as the decrease in non-aerated lung volumes:

Alveolar recruitment (mL) = (CMV non-aerated lung volume (mL))–(HFPV non-aerated lung volume (mL)).

HFPV tidal volume was defined by volume difference between end-inspiratory and end-expiratory holds.

### Animal experiments

This study was approved by the National Ethics Committee on animal research (approval number 01505.01), and was carried out in accordance with the International Guiding Principles for Biomedical Research Involving Animals [24].

Briefly, after general anesthesia induction, animals were equipped with right lateral thoracic surgical drainage (Seldinger Chest Drainage Kit, Portex®, Smith Medical), to monitor pleural pressure (Ppl). A rigid 30-cm-long catheter was inserted into the endotracheal tube (diameter 8) towards the distal lumen to monitor tracheal upper airways pressure (Paw). Injurious mechanical ventilation was completed with hydrochloric acid tracheal instillation to model severe ARDS as adapted from Ambrosio [25]. Animals were ventilated with VDR-4 with random pressure levels.

#### Statistical analysis

Categorical data were expressed as numbers and percentages, and quantitative data as mean (SD) or median (IQR). Distribution normality was tested using the Shapiro-Wilk test. Continuous variables analyses were performed using Student’s *t* test or the non-parametric Mann-Whitney *U* test. Changes in arterial blood gases and hemodynamic data were analyzed by longitudinal analysis using mixed models to take into account between-subject and within-subject variability (with patient as random effect). Spearman’s rank correlation coefficient was calculated to assess the relationship between HFPV-induced hyperinflation and CMV volumes, and animal pulmonary pressures monitoring. Monitored and measured pressure was compared by Bland and Altman analysis for multiple measurements. *P* < 0.05 (two-sided) was considered significant.

## Results

### Study patients

Between February and July 2015, eight patients with moderate to severe non-focal ARDS were enrolled within 24 hours of disease onset. Table 1 summarizes the baseline characteristics.

**Table 1:** Characteristics of patients

Patient number	Etiology	Comorbidity	IBW (kg)	ARDS onset (h)	FiO_2_ (%)	PaO_2_/FiO_2_ (mmHg)	PEEP (cmH2O)	Tidal volume (mL/kg IBW)	Compliance (mL.cmH_2_O^-1^)	Pplat (cmH2O)	ARDS phenotype
1	Infectious Pneumonia	Acute leukemia	63	18	50	154	8	7.6	20	33	NF
2	Infectious Pneumonia	Myelofibrosis	70	6	100	127	16	5.9	25	33	NF
3	Infectious Pneumonia	Acute leukemia	55	24	70	109	18	5.8	21	33	NF
4	Infectious Pneumonia		47	4	100	75	12	5.7	13	28	NF
5	Infectious Pneumonia	Acute leukemia	54	10	60	145	13	6.3	18	31	NF
6	Aspiration	Cardiac arrest	73	4	100	50	12	5 .8	17	30	NF
7	Aspiration	Gastrectomy	77	12	100	80	18	5.5	20	28	NF
8	Viral pneumonia		64	6	70	113	20	7.4	18	32	NF
Median			63.5	8.0	85	111	14.5	5.9	19.0	31.5	
IQR			(55.0–70.8)	(5.5–13.5)	(68–100)	(79–132)	(12.0–18.0)	(5.8–6.6)	(17.8–20.3)	(29.5–33.0)	

Data are presented as median (IQR). *Abbreviations*: *ARDS* acute respiratory distress syndrome, *IBW* ideal body weight (Lorentz formula), *FiO*_*2*_ fraction of inspired oxygen, *PaO*_*2*_ arterial oxygen tension, *NF* non-focal, *PEEP* positive end-expiratory pressure, *Pplat* plateau pressure

Prior to inclusion, mean CMV time, arterial oxygen tension (PaO_2_)/fraction of inspired oxygen (FiO_2_) ratio and tidal volume (Vt) were 8 (4.5–16.5) hours, 111 (76–141) mmHg and 5.9 (5.8–7.1) mL.kg^-1^ of ideal body weight (IBW), respectively. PEEP and plateau pressures were respectively 14.5 (12.0–18.0) and 31.5 (28.5–33.0) cmH_2_0. Lung static compliance was 20.5 (17.3–36.3) mL.cmH_2_O^-1^. Lung morphology was exclusively non-focal. HFPV settings are presented in Additional file 1: Table S1.

#### Changes in hemodynamics and respiratory parameters

**Fig. 1:**
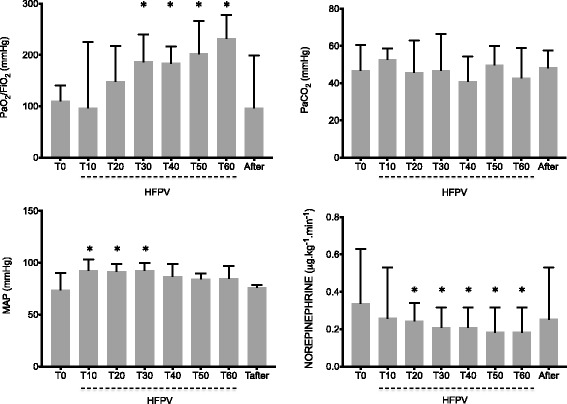
Evolution of arterial oxygen tension (PaO_2_) to inspiratory oxygen fraction (FiO_2_) ratio and arterial carbon dioxide tension (PaCO_2_) (*upper graphs*) and hemodynamic parameters (*lower graphs*): Mean arterial pressure (MAP) and Norepinephrine doses during the experimental procedure. *P < 0.05 versus time 0 (T0). *HFPV* high frequency percussive ventilation

Arterial blood gases showed substantial increase in PaO_2_/FiO_2_ 30 minutes after HFPV initiation (T30), which persisted while under HFPV (Fig. 1a). Oxygenation benefits disappeared after resuming CMV (Fig. 1a, T-after). No PaCO_2_ change was observed (Fig. 1b).

Mean arterial pressure (MAP) increased significantly between T10 and T30 (Fig. 1c) while norepinephrine doses dropped from 0.34 (0.24–0.63) (T0) to 0.19 (0.08–0.32) μg.kg^-1^.min^-1^ (T60, Fig. 1d). Benefits disappeared after resuming CMV (0.26 (0.04–0.53) μg.kg^-1^.min^-1^, T-after, Fig. 1d). Vasopressor index (VI) and vasopressor dependency index [26] followed similar trends (Additional file 1: Figure S1).

A significant increase in lung compliance was observed (T-after, 28.5 (24.5–32.3) versus T0, 20.5 (17.8–28.8) mL.cmH_2_O^-1^, *P* = 0.04), without any airway resistance change (13.5 (12.5–14.0) versus 14.5 (11.0–15.5) mL.cmH_2_O^-1^, *P* = 0.58, T-after vs T0, respectively). (Additional file 1: Table S2).

#### CT scan analysis

Tidal volumes remained stable over treatment duration: 5.9 (5.8–7.1) versus 5.7 (1.1–8.1) versus 6.1 (5.8–6.2) mL.kg^-1^ ideal body weight (IBW) (T0 versus HFPV versus T-after, *P* = 0.90). Alveolar recruitment, defined as the change in non-aerated lung volumes, increased significantly between CMV and HFPV (12.0% (8.5–18.0), *P =* 0.05 and 12.5% (9.3–16.8), *P* = 0.003, end-expiratory and end-inspiratory holds, respectively, Additional file 1: Table S3). When recruitment was assessed as the change in non-aerated and poorly aerated lung volumes, no significant evolution was observed between CMV and HFPV (12.0% (5.0–18.0) with end-expiratory hold, *P =* 0.27 and 11.0% (6.0–22.0) with end-inspiratory hold, *P* = 0.10, Additional file 1: Table S3).

**Fig. 2:**
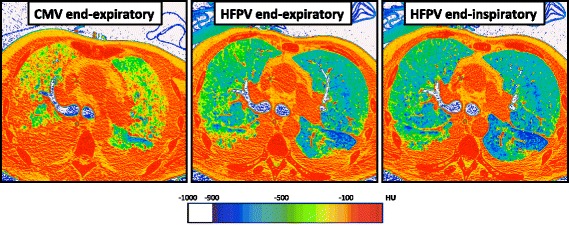
UCLA color encoding of lung computed tomography (CT) attenuation in a patient with non-focal acute respiratory distress syndrome (ARDS) phenotype. Direct visualization of lung aeration was performed after processing CT scan images with CT attenuation color-encoding. In this patient with non-focal ARDS, high frequency percussive ventilation (HFPV) resulted in an important recruitment of non-aerated (red) lung zones, and increasing normally aerated (blue) ones. HFPV allowed large alveolar recruitment and was associated with almost no concomitant hyperinflation (white) of aerated lung regions. Consecutive images were recorded using: (1) an end-expiratory hold during conventional mechanical ventilation, (2) an end-expiratory hold or (3) an end-inspiratory hold during HFPV. Color encoding of CT attenuation: hyperinflation (white), normal aeration (blue), poor aeration (green) and absent aeration (red). *CMV* conventional mechanical ventilation

No significant change in hyperinflated lung volumes was observed: 2.0% (0.5–2.5) (CMV versus HFPV end-expiratory hold, *P =* 0.89) and 3.0% (2.5–4.00 (CMV versus HFPV end-inspiratory hold, *P =* 0.27) (Fig. 2 and Additional file 1: Table S3)*.* Complete changes in lung volumes and masses are presented in Additional file 1: Table S4. Global increases in end-expiratory lung volume (EELV), total lung volume and normally-aerated lung volumes were observed.

Evolution of lung volumes’ absolute ratios according to aeration status and West zone are presented in Additional file 1: Figures S2 and S3. End-expiratory non-aerated volumes significantly decreased especially in posterior (dependant) lung zones. Normally aerated lung volumes increased in posterior lung regions. Hyperinflated and poorly aerated lung volumes remained stable. HFPV end-expiratory hold hyperinflated lung volume was correlated with CMV normally aerated (*r =* 0.79, *P =* 0.05) and hyperinflated (*r =* 0.79, *P =* 0.05) lung volumes. HFPV end-inspiratory hold hyperinflated lung volume was correlated with CMV hyperinflated lung volumes (*r =* 0.88, *P =* 0.01) (Additional file 1: Table S4).

#### Animal experiments

**Fig. 3:**
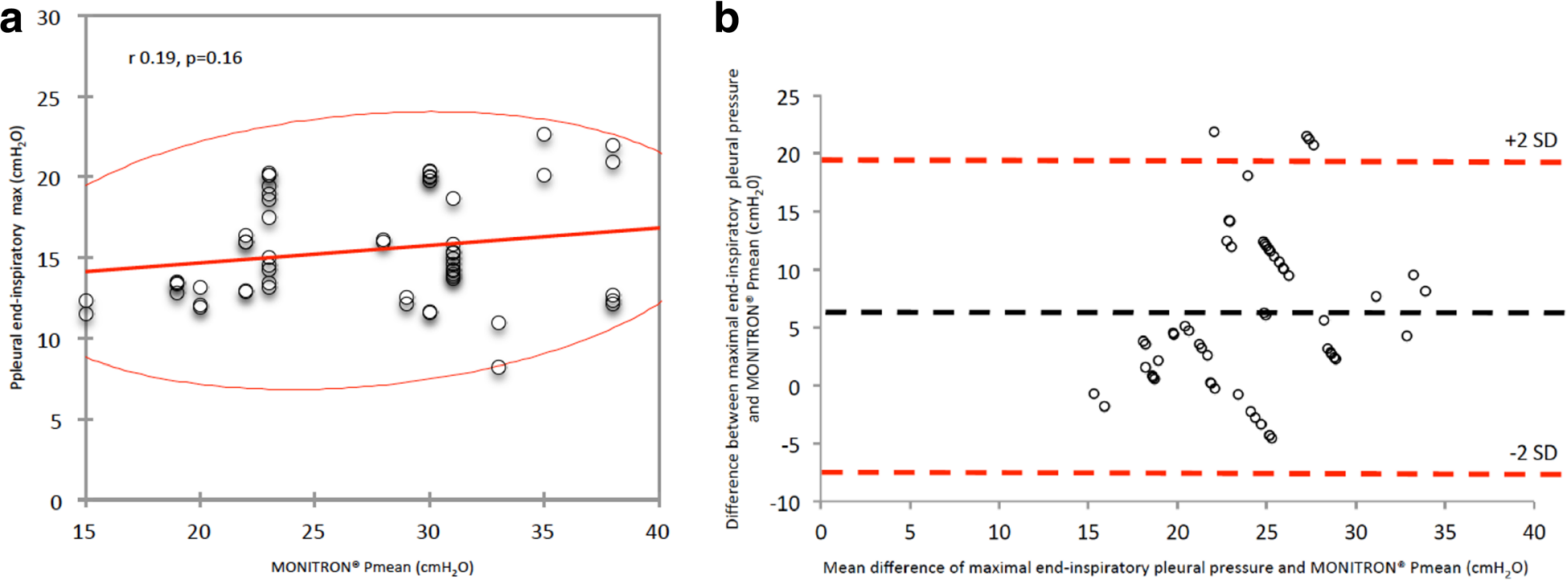
Correlation and Bland and Altman bias between maximal end-inspiratory pleural pressure and high frequency percussive ventilation (HFPV) mean pressures considering all pairs of measurements performed during the study. **a** *N* = 58, red line: 95% confidence ellipsis; **b** *N* = 58, lines: bias (black dotted) and +2SD/-2SD limits of agreement (red dotted). *SD* standard deviation

Additional file 1: Table S5 presents correlations and bias between Ppl or Paw and absolute values of Monitron®-derived PEEP, mean and peak pressures. Figure 3 presents graphic illustration of interactions between maximal end-inspiratory Ppl and Monitron®-derived mean pressure. When considering the 58 pairs of measurements, bias (lower to upper limits of agreement) between absolute values of maximal end-inspiratory Ppl and HFPV mean pressure was 6.1 (−7.3 to 19.5) cmH_2_O, without any correlation (*r* = 0.19, *P =* 0.16). Maximal end-inspiratory Ppl remained below 25cmH_2_O despite elevated HFPV mean pressures. Further analyses and correlations are presented in Additional file 1.

### Discussion

This study is, to our knowledge, the first to demonstrate HFPV effects on lung aeration and tidal volume. Significant alveolar recruitment and tidal volume close to 6 ml/kg IBW were observed in patients with early non-focal ARDS receiving short-term HFPV. This study is also the first to investigate in vivo correlation between Phasitron and transpulmonary pressures.

### Alveolar recruitment

**Fig. 4:**
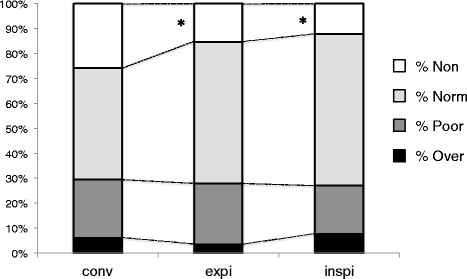
Evolution of lung volumes under conventional ventilation and high frequency percussive ventilation (HFPV). *Abbreviations*: *conv* conventional ventilation, *expi* expiratory hold during HFPV, *inspi* inspiratory hold during HFPV, *Non* non-aerated lung volume, *Norm* normally aerated lung volume, *Over* overdistended lung volume, *Poor* poorly aerated lung volume. Data are presented as percentages of total lung volume. *P < 0.05 versus conventional ventilation

Alveolar recruitment was statistically significant. Under CMV, alveolar recruitment is induced either by PEEP incremental increases and tidal recruitment (*e-sigh*) or transpulmonary pressure transient increase [27]. Although alveolar recruitment remains difficult to assess at the bedside [28], it can be measured by analysis of lung CT scans [22]. The effects of RM on lung morphology have already been reported [23], and alveolar recruitment (poorly aerated and non-aerated volumes decrease) [29] was effective in both focal and non-focal ARDS following an RM (40 cmH_2_O, 40 seconds). Recruitment reached 6 ± 6% and 18 ± 8% in focal and non-focal ARDS (*P =* 0.004) [23], whereas we observed recruitment gains in around 12% depending on alveolar recruitment definition and HFPV end-expiratory or end-inspiratory holds (Additional file 1: Table S3). High frequency mini-bursts may allow a progressive move from alveolar collapse to re-opening, through discrete jumps [30]. The pragmatic definition of lung recruitment that we used clearly depicts HFPV effects over CMV in terms of alveolar recruitment. Indeed, no significant change in poorly aerated volumes was observed (Fig. 4). Lung recruitment seems predominant in posterior zones where non-aerated volumes are observed. Of note, CMV preliminary optimization and absence of zero-PEEP end-expiratory CT scan may have conducted to lesser HFPV effects on alveolar recruitment.

### Alveolar hyperinflation

Alveolar hyperinflation is often associated with alveolar recruitment since intra-thoracic pressure increase may preferentially inflate normally aerated (high compliance) more than non-aerated (low compliance) lung regions, if there is lung inhomogeneity. No difference was observed between HFPV-induced and CMV-induced hyperinflation, in line with a previous report [31]. Hyperinflation was significantly correlated with baseline normally aerated and hyperinflated lung volumes, whereas our group reported a single correlation with normally aerated ones [23]. Those results agree with previous observations from Terragni, who reported that tidal hyperinflation persists despite protective ventilation [32]. Of particular interest in focal ARDS, high PEEP levels might sustain hyperinflation of non-dependent lung regions [33], as observed. Moreover, tidal recruitment/de-recruitment might worsen the patient’s status by exposing lung regions to shear stress [34], as observed in focal ARDS. Those observations were negligible in non-focal ARDS. Early identification of lung morphology should be of the highest importance in adapting mechanical ventilation strategies [35]. HFPV was suggested to better adapt Vt and lung pressures to dynamic changes in gas distribution [36], thus hampering prediction of its pathophysiological effects.

In our study, hyperinflation remained stable within all three West zones. As previously reported [23, 35], low hyperinflated lung regions have been observed while performing an RM, when dealing with a non-focal ARDS phenotype (in comparison to focal ARDS).

Finally, HFPV use as RM and open-lung strategy surrogates remains uncertain. RM has been reported to increase hyperinflation by 8 ± 9% and 24 ± 14% in patients with non-focal and focal ARDS [23]. In our study, hyperinflation was negligible in non-focal ARDS, at 2.0% (1.0–4.0).

### Tidal volume

The difference between end-inspiratory and end-expiratory volumes approximated the Vt. HFPV-induced Vt remained within recommended limits. This observation supports an HFPV lung-protective ventilation strategy. Vt measurement on CT scans might be affected by oxygen consumption and stress relaxation. However, acquisition lasted a few seconds and was similar between both holds.

### Oxygenation

HFPV markedly improved oxygenation. HFPV-induced alveolar recruitment may improve gas exchange surface and decrease ventilation/perfusion mismatch. Also, effective gas volume administered does not only include Vt but also volume from high frequency mini-bursts. Lucangelo showed in a single compartment lung model, that Vt accounts for approximately 10–40% of total administered volume [36]. Diffusive ventilation may therefore allow large inflow and out-flow, thus increasing alveolar gas mixing (O_2_ in and CO_2_ out).

### Hemodynamics

Hemodynamics improved significantly during HFPV, the effect remaining unclear. As compared to RM-induced transient increase in intra-thoracic pressure, HFPV treatments did not decrease arterial pressure or induce serious adverse hemodynamic events. HFPV may reduce shunts through improved alveolar recruitment, and enhance peripheral arterial vasoconstriction by improving arterial oxygenation [37, 38]. In addition, HFPV may rapidly increase alveolar recruitment, lung aeration and arterial oxygenation, thus improving pulmonary vascular function and facilitating right ventricle output.

### Monitoring of pressure

Monitron® device monitored high pressure during HFPV. Phasitron®-delivered pressures (recorded before endotracheal tube) are higher and correlate with Ppl. Intra-thoracic and transpulmonary pressures evaluations [39, 40] are relevant tools in understanding HFPV effects. Careful measurement of Ppl through the pleural drain and Paw through the intra-tracheal catheter allowed useful comparisons. We found good correlation (despite large agreements limits) between the Monitron® and measured pressures. Of high interest, Ppl remained within the protective limits: alveolar pressure was always below 30 cmH_2_O (and especially, below 25cmH_2_O in our experiments) whatever the Monitron® pressure, even with apparently excessive pressure settings. This 30-cmH_2_0 pressure seems to limit the risk of distension-related lung injury as reported by Boussarsar [41] and Tobin [42]. A recent study elucidated pressure drops through endotracheal tubes [43]. During the mini-burst inspiratory phase, pressure drops were: 9.28 (4.95–12.93), 9.48 (5.05–13.47), and 10.04 (5.62–16.97) cmH_2_O for diameters 8, 7.5 and 6.5, respectively [44]. Administered pressures are strongly dampered through the endotracheal tube and airways. This phenomenon might explain low measured Ppl, well below the Monitron®-specified ones.

#### Limitations

Our study has several limitations. First, only eight patients were enrolled. Results presented herein only reflect HPFV effects on the lungs of patients with non-focal early ARDS. This could limit generalizability and/or dampen the strength of current associations. Further studies on patients with focal ARDS are necessary. Nevertheless, we believe that our findings represent an important first step in vivo to elucidate HFPV effects on the lungs in early non-focal ARDS. Second, hemodynamic improvements remain unexplained since no specific monitoring was used in our study, and further investigations are warranted. Third, only patients with early ARDS were included and extrapolation to later phases of ARDS remains uncertain. Fourth, CMV was optimized following “open-lung” ventilation recommendations; to what extent ventilator settings may have influenced our results remains unknown. Indeed, the Express study ventilator settings and strategy are supposed to optimize alveolar recruitment. No comparison with RM strategies was conducted and alveolar recruitment maximization remains empirical. Finally, the absence of a zero end-expiratory CT scan could have underestimated patients at risk of tidal hyperinflation, but remains delicate to be proposed to severe-to-moderate ARDS patients.

## Conclusions

This study elucidates HFPV morphological effects in patients with early non-focal ARDS. Besides impressive improvements in arterial oxygenation and hemodynamics, HFPV-induced alveolar recruitment was significant. Therefore, HFPV might be used as a rescue therapy in early non-focal ARDS patients when CMV fails to improve oxygenation and lung aeration. However, further investigations are needed to assess HFPV effects on patient outcomes during ARDS, with a special focus on focal and non-focal lung phenotypes.

## Additional file

Additional file 1: Supplementary methods, figures and tables. (DOCX 2972 kb)

## Abbreviations

ARDS: Acute respiratory distress syndrome
CMV: Conventional mechanical ventilation
CT: Computed tomography
EELV: End-expiratory lung volume
FiO_2_: Fraction of inspired oxygen
HFO: High frequency oscillation
HFPV: High frequency percussive ventilation
HU: Hounsfield unit
IBW: Ideal body weight
ICU: Intensive Care Unit
IQR: Inter-quartile range
MAP: Mean arterial pressure
OLV: Open lung ventilation
PaO_2_: Arterial oxygen tension
Paw: Upper airways pressure
PEEP: Positive end-expiratory pressure
RM: Recruitment manoeuver
SD: Standard deviation
VDR: Volumetric diffusive respirator
VI: Vasopressor index
VILI: Ventilator induced lung injury
Vt: Tidal volume

## References

[1] Slutsky AS, Ranieri VM (2013). Ventilator-induced lung injury. N Engl J Med.

[2] Network ARDS, Brower RG, Matthay MA, Morris A, Schoenfeld D, Thompson BT, Wheeler A (2000). Ventilation with lower tidal volumes as compared with traditional tidal volumes for acute lung injury and the acute respiratory distress syndrome. N Engl J Med.

[3] Mercat A, Richard JC, Vielle B, Jaber S, Osman D, Diehl JL, Lefrant JY, Prat G, Richecoeur J, Nieszkowska A (2008). Positive end-expiratory pressure setting in adults with acute lung injury and acute respiratory distress syndrome: a randomized controlled trial. JAMA.

[4] Guerin C, Reignier J, Richard JC, Beuret P, Gacouin A, Boulain T, Mercier E, Badet M, Mercat A, Baudin O (2013). Prone positioning in severe acute respiratory distress syndrome. N Engl J Med.

[5] Damiani LP, Berwanger O, Paisani D, Laranjeira LN, Suzumura EA, Amato MBP, Carvalho CRR, Cavalcanti AB (2017). Statistical analysis plan for the Alveolar Recruitment for Acute Respiratory Distress Syndrome Trial (ART). A randomized controlled trial. Rev Bras Ter Intensiva.

[6] Ferguson ND, Cook DJ, Guyatt GH, Mehta S, Hand L, Austin P, Zhou Q, Matte A, Walter SD, Lamontagne F (2013). High-frequency oscillation in early acute respiratory distress syndrome. N Engl J Med.

[7] Young D, Lamb SE, Shah S, MacKenzie I, Tunnicliffe W, Lall R, Rowan K, Cuthbertson BH, Group OS (2013). High-frequency oscillation for acute respiratory distress syndrome. N Engl J Med.

[8] Rizkalla NA, Dominick CL, Fitzgerald JC, Thomas NJ, Yehya N (2014). High-frequency percussive ventilation improves oxygenation and ventilation in pediatric patients with acute respiratory failure. J Crit Care.

[9] Allan PF, Osborn EC, Chung KK, Wanek SM (2010). High-frequency percussive ventilation revisited. J Burn Care Res.

[10] Chung KK, Wolf SE, Renz EM, Allan PF, Aden JK, Merrill GA, Shelhamer MC, King BT, White CE, Bell DG (2010). High-frequency percussive ventilation and low tidal volume ventilation in burns: a randomized controlled trial. Crit Care Med.

[11] Cartotto R, Ellis S, Gomez M, Cooper A, Smith T (2004). High frequency oscillatory ventilation in burn patients with the acute respiratory distress syndrome. Burns.

[12] Reper P, Van Bos R, Van Loey K, Van Laeke P, Vanderkelen A (2003). High frequency percussive ventilation in burn patients: hemodynamics and gas exchange. Burns.

[13] Eastman A, Holland D, Higgins J, Smith B, Delagarza J, Olson C, Brakenridge S, Foteh K, Friese R (2006). High-frequency percussive ventilation improves oxygenation in trauma patients with acute respiratory distress syndrome: a retrospective review. Am J Surg.

[14] Dmello D, Nayak RP, Matuschak GM (2010). High-frequency percussive ventilation for airway clearance in cystic fibrosis: a brief report. Lung.

[15] Lucangelo U, Zin WA, Fontanesi L, Antonaglia V, Peratoner A, Ferluga M, Marras E, Borelli M, Ciccolini M, Berlot G (2012). Early short-term application of high-frequency percussive ventilation improves gas exchange in hypoxemic patients. Respiration.

[16] Blondonnet R, Aliane J, Godet T, Souweine B, Constantin JM (2015). High-frequency percussive ventilation as a rescue therapy for ARDS patients under ECMO: about a case. Anaesth Crit Care Pain Med.

[17] Boscolo A, Peralta A, Baratto F, Rossi S, Ori C (2015). High-frequency percussive ventilation: a new strategy for separation from extracorporeal membrane oxygenation. A A Case Rep.

[18] Salim A, Martin M (2005). High-frequency percussive ventilation. Crit Care Med.

[19] Flow Ventilation(R). https://percussionaire.com/flow-ventilation/. Accessed Apr 2017.

[20] Godet T, Jabaudon M, Blondonnet R, Pereira B, Garcier J-M, Futier E, Constantin J-M. High frequency percussive ventilation increases alveolar recruitment in patients with early acute respiratory distress syndrome. A physiological CT-scan study. In: ATS 2016. vol. 193. San Francisco: Am J Respir Crit Care Med. 2016. 10.1186/s13054-017-1924-6 PMC5763966 29325586

[21] Force ADT, Ranieri VM, Rubenfeld GD, Thompson BT, Ferguson ND, Caldwell E, Fan E, Camporota L, Slutsky AS (2012). Acute respiratory distress syndrome: the Berlin Definition. JAMA.

[22] Puybasset L, Cluzel P, Gusman P, Grenier P, Preteux F, Rouby JJ (2000). Regional distribution of gas and tissue in acute respiratory distress syndrome. I. Consequences for lung morphology. CT Scan ARDS Study Group. Intensive Care Med.

[23] Constantin JM, Grasso S, Chanques G, Aufort S, Futier E, Sebbane M, Jung B, Gallix B, Bazin JE, Rouby JJ (2010). Lung morphology predicts response to recruitment maneuver in patients with acute respiratory distress syndrome. Crit Care Med.

[24] International guiding principles for biomedical research involving animals issued by CIOMS. Vet Q. 1986; 8(4):350-2. 10.1080/01652176.1986.9694068 22050297

[25] Ambrosio AM, Luo R, Fantoni DT, Gutierres C, Lu Q, Gu WJ, Otsuki DA, Malbouisson LM, Auler JO, Rouby JJ (2012). Effects of positive end-expiratory pressure titration and recruitment maneuver on lung inflammation and hyperinflation in experimental acid aspiration-induced lung injury. Anesthesiology.

[26] Cruz DN, Antonelli M, Fumagalli R, Foltran F, Brienza N, Donati A, Malcangi V, Petrini F, Volta G, Bobbio Pallavicini FM (2009). Early use of polymyxin B hemoperfusion in abdominal septic shock: the EUPHAS randomized controlled trial. JAMA.

[27] Chiumello D, Algieri I, Grasso S, Terragni P, Pelosi P. Recruitment maneuvers in acute respiratory distress syndrome and during general anesthesia. Minerva Anestesiol. 2016;82(2):210–20. Epub 2015 Apr 17. 25881732

[28] Godet T, Constantin JM, Jaber S, Futier E (2015). How to monitor a recruitment maneuver at the bedside. Curr Opin Crit Care.

[29] Malbouisson LM, Muller JC, Constantin JM, Lu Q, Puybasset L, Rouby JJ, Group CTSAS (2001). Computed tomography assessment of positive end-expiratory pressure-induced alveolar recruitment in patients with acute respiratory distress syndrome. Am J Respir Crit Care Med.

[30] Suki B, Barabasi AL, Hantos Z, Petak F, Stanley HE (1994). Avalanches and power-law behaviour in lung inflation. Nature.

[31] Borges JB, Okamoto VN, Matos GF, Caramez MP, Arantes PR, Barros F, Souza CE, Victorino JA, Kacmarek RM, Barbas CS (2006). Reversibility of lung collapse and hypoxemia in early acute respiratory distress syndrome. Am J Respir Crit Care Med.

[32] Terragni PP, Rosboch G, Tealdi A, Corno E, Menaldo E, Davini O, Gandini G, Herrmann P, Mascia L, Quintel M (2007). Tidal hyperinflation during low tidal volume ventilation in acute respiratory distress syndrome. Am J Respir Crit Care Med.

[33] Nieszkowska A, Lu Q, Vieira S, Elman M, Fetita C, Rouby JJ (2004). Incidence and regional distribution of lung overinflation during mechanical ventilation with positive end-expiratory pressure. Crit Care Med.

[34] Grasso S, Terragni P, Mascia L, Fanelli V, Quintel M, Herrmann P, Hedenstierna G, Slutsky AS, Ranieri VM (2004). Airway pressure-time curve profile (stress index) detects tidal recruitment/hyperinflation in experimental acute lung injury. Crit Care Med.

[35] Grasso S, Stripoli T, Sacchi M, Trerotoli P, Staffieri F, Franchini D, De Monte V, Valentini V, Pugliese P, Crovace A (2009). Inhomogeneity of lung parenchyma during the open lung strategy: a computed tomography scan study. Am J Respir Crit Care Med.

[36] Lucangelo U, Antonaglia V, Zin WA, Berlot G, Fontanesi L, Peratoner A, Bernabe F, Gullo A (2006). Mechanical loads modulate tidal volume and lung washout during high-frequency percussive ventilation. Respir Physiol Neurobiol.

[37] Spoelstra-de Man AM, Smit B, Oudemans-van Straaten HM, Smulders YM (2015). Cardiovascular effects of hyperoxia during and after cardiac surgery. Anaesthesia.

[38] Moradkhan R, Sinoway LI (2010). Revisiting the role of oxygen therapy in cardiac patients. J Am Coll Cardiol.

[39] Gattinoni L, Chiumello D, Carlesso E, Valenza F (2004). Bench-to-bedside review: chest wall elastance in acute lung injury/acute respiratory distress syndrome patients. Crit Care.

[40] Chiumello D, Cressoni M, Colombo A, Babini G, Brioni M, Crimella F, Lundin S, Stenqvist O, Gattinoni L (2014). The assessment of transpulmonary pressure in mechanically ventilated ARDS patients. Intensive Care Med.

[41] Boussarsar M, Thierry G, Jaber S, Roudot-Thoraval F, Lemaire F, Brochard L (2002). Relationship between ventilatory settings and barotrauma in the acute respiratory distress syndrome. Intensive Care Med.

[42] Tobin MJ (2000). Culmination of an era in research on the acute respiratory distress syndrome. N Engl J Med.

[43] Ajcevic M, Lucangelo U, Ferluga M, Zin WA, Accardo A (2014). In vitro estimation of pressure drop across tracheal tubes during high-frequency percussive ventilation. Physiol Meas.

[44] Ajcevic M (2014). Personalized setup of high frequency percussive ventilator by estimation of respiratory system viscoelastic parameters.

