# Supplementary material for: High frequency percussive ventilation increases alveolar recruitment in early acute respiratory distress syndrome: an experimental, physiological and CT scan study
Source: Crit Care. 2018 Jan 11;22:3. doi: 10.1186/s13054-017-1924-6 (PMC5763966; doi:10.1186/s13054-017-1924-6)
Supplement: Additional file 1: — Supplementary methods, figures and tables. (DOCX 2972 kb) [file 13054_2017_1924_MOESM1_ESM.docx]

**ADDITIONAL FILE**

**High Frequency Percussive Ventilation increases alveolar recruitment in early acute respiratory distress syndrome**

A physiological CT-Scan study

Thomas Godet, MD, PhD, Matthieu Jabaudon, MD, PhD, Raïko Blondonnet, MD, MSc, Aymeric Tremblay, MD, Jules Audard, MSc, Benjamin Rieu, MSc, Bruno Pereira, PhD, Jean-Marc Garcier, MD, PhD, Emmanuel Futier, MD, PhD, Jean-Michel Constantin, MD, PhD

**Materials and Methods**

*ARDS definition*

ARDS was defined following Berlin’s definition: bilateral opacities consistent with pulmonary edema must be present and may be detected on CT or chest radiograph, a PaO_2_/FiO_2_ ratio below 300mmHg with a minimum of 5 cmH_2_0 PEEP (or CPAP) and must not be  fully explained by cardiac failure or fluid overload. (1)

*Mechanical ventilation*

Conventional mechanical ventilation (CMV) of patients was optimized and total duration lasted few hours (< 24 hours) prior to inclusion. CMV followed « open lung » and protective ventilation international guidelines. Tidal volume target was 6 mL.kg^-1^ of ideal body weight. Plateau pressure was maintained below 30 cmH_2_O. FiO2 was the lowest permitting a SpO2 between 88 and 92% or a PaO2 around 60 mmHg. Respiratory rates were set below 35.min^-1^, and adjusted to obtain a pH > 7.30, a PaCO2 in the range 35-40 mmHg and to avoid gas trapping. Positive end-expiratory pressure was set according to Express study table (group optimized recruitment).(2) Mechanical ventilation was provided using an Engström Carestation (General Electrics Healthcare).

Patients were ventilated with HFPV using VDR-4 (Volumetric Diffusive Respirator, Percussionaire® Corporation, Bird Technologies, Sandpoint, ID) as a stand-alone ventilator, with a mandatory maximum 30 cmH_2_O mean pressure and a CMV equivalent PEEP. Adjustments were obtained following manufacturer’s recommendations. Frequencies were set at 600.min^-1^ for mini-bursts and to obtain end tidal CO_2_ equivalent to the one during CMV, for convective frequency. The following picture illustrates pressure waveform during HFPV as given by Monitron® analyzer.


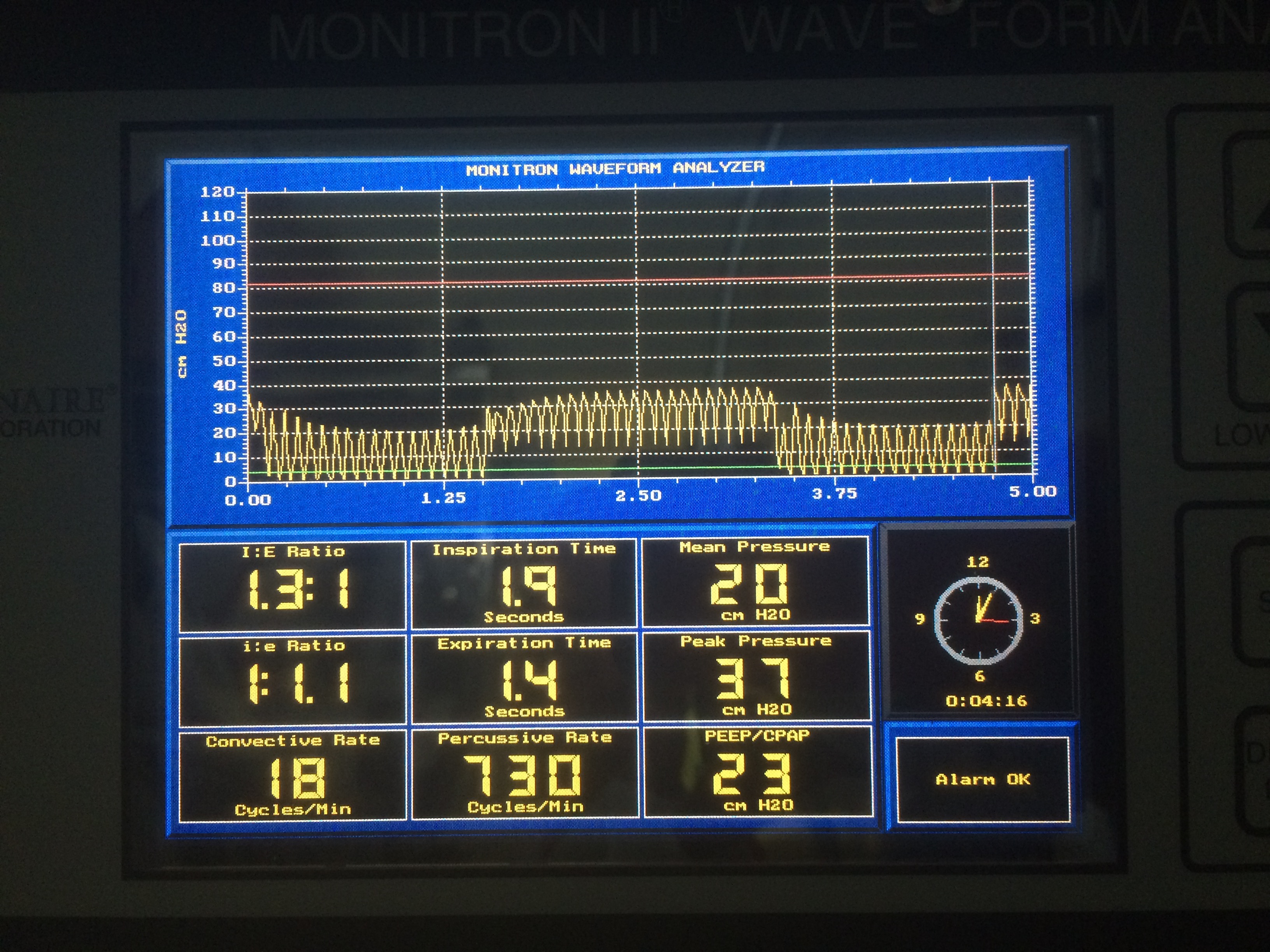


Adequate humidification was assured by a high-volume nebulizer incorporated in the ventilator circuit and an external heated humidifier (F&P 850™ System; Fisher and Paykel Health-care, Auckland, NZ) either during CMV or HFPV.

*Hemodynamic optimization*

Volume optimization followed trans-thoracic echocardiography data (left ventricle filling pressures according to mitral doppler E/A and E/Ea) and eventually hemodynamic responses to previous recruitment maneuver. If necessary, fluid loading with NaCl 0.9% was titrated by bolus of 250 mL. Norepinephrine infusion was introduced to maintain mean arterial pressure above 70 mmHg.

*Intravenous medications*

Anesthesia was performed with continuous infusions of propofol and remifentanil. Sedation was monitored according to frontal bispectral Index (target 40-60). Neuromuscular blockade was obtained with continuous infusion of cisatracurium and monitored to obtain a train of four below 2.

*Transportation to radiology*

Each patient was transported to the Department of Radiology (Estaing Hospital, University Hospital of Clermont-Ferrand) by two senior intensivists (TG and JMC). Mechanical ventilation was provided using an Engström Carestation (General Electrics Healthcare) delivering 100% oxygen. During transport, CMV was provided by the same ventilator used for clinical purposes with unchanged parameters. Heart rate, pulse oxymetry and systemic arterial pressure were monitored continuously using a Philips IntelliVue MMS X2 (Eindhoven, The Netherlands).

*CT scan*

Lung scanning was performed in the supine position from the apex to the lung base using a multidetector computed tomography (Discovery CT750 HD, General Electrics Healthcare), without iodine contrast media injection. Contiguous axial CT sections of 0.625 mm thick of lung parenchyma were reconstructed from the volumetric data. CT scans were obtained at 100kV and 600mA.

First acquisition of spiral CT sections was performed at end-expiratory hold by clamping endotracheal tube under conventional mechanical ventilation. Second and third acquisitions were performed at end-expiratory and end-inspiratory holds, respectively, under high frequency percussive ventilation. Whole-lung acquisitions lasted less than 4 seconds.

No preliminary CT scan in ZEEP (zero end-expiratory pressure) was obtained due to large risk of oxygen desaturation in most patients and considered unethical due to illness severity.

**Figure S0**. Timeline of experimental protocol. *Definition of abbreviations*: CMV: conventional mechanical ventilation; conv: end-expiratory hold during conventional mechanical ventilation; CT: computed tomography;expi: end-expiratory hold during high frequency percussive ventilation; HFPV: high frequency percussive ventilation; ICU: intensive care unit; inspi: end-inspiratory hold during high frequency percussive ventilation.

*Images post-processing*

CT-scans of lung parenchyma were computed to obtain 5 mm thick contiguous sections (DICOM, Advance Workstation, General Electrics Healthcare). All images were observed at a window width of 1,600 HU and a level of -700 HU.

To allow qualitative assessment of lung tissue aeration, images were read based on the UCLA color-coding table (<http://osirixfoundation.com>; OsiriX image processing software, OsiriX, Geneva, Switzerland).

Quantitative assessment of lung morphology was performed using dedicated software (Maluna 3.17, University Hospital of Göttingen, Germany). Regions of Interest (ROI) were drawn manually including only lung parenchyma and excluding large vessels and bronchi. Lung gas content (*i.e.* functional residual capacity, FRC), lung weight and distribution of aeration were computed as previously reported (3). Aeration of lung tissue was divided into four compartments according to their Hounsfield Units (HU): hyperinflated tissue (densities from -1000 to -900 HU), normally aerated tissue (-900 to -500 HU), poorly aerated tissue (-500 to -100 HU) and non-aerated tissue (-100 to 100 HU). CT-scans and manual drawings were reviewed by 2 ICU physicians (TG and JMC) and an expert radiologist (JMG). Interobserver agreements were good (*κ*=0.82). Maluna analysis of each CT scan required at least 5 hours, for a trained investigator.

Recruitment was calculated either during inspiratory and expiratory holds as the decrease in non-aerated lung volumes following HFPV:

Alveolar recruitment (mL) = Non-aerated lung volume (mL) under conventional ventilation – Non-aerated lung volume (mL) after HFPV.

Tidal volume was evaluated as the difference between end-inspiratory and end-expiratory holds:

Tidal Volume (mL) = End-inspiratory lung volume (mL) – End-expiratory lung volume (mL).

*Definitions of hemodynamic parameters (4)*

The dose of vasoactive/vasopressor agents is expressed as the vasopressor index (VI), a dimensionless variable calculated as:

VI = (dopamine dose x 1) + (dobutamine dose x 1) + (adrenaline dose x 100) +

(noradrenaline dose x 100) + (phenylephrine dose x 100)

wherein all doses are expressed as μg.kg^-1^.min^-1^.

In clinical practice, the vasopressor dose is titrated periodically according to the blood pressure. Therefore, a dose-response relationship between vasopressor dose and MAP was used as another surrogate for the degree of hemodynamic impairment. This dose- response relationship was expressed as the vasopressor dependency index, which is calculated as the ratio of vasopressor index to MAP; the higher the score, the greater the vasopressor requirement.

*Patients’ radiation exposure*

Mean value of X-rays exposure was 1242±178 mGy.cm^-1^ during full protocol.

*Statistics*

Analyses were performed using XLSTAT^©^ Version 2016.05.35073 (Addinsoft, Paris, France) and PRISM^©^ Version 7.0b (GraphPad Software, Inc). Due to the low number of patients in the focal group, no statistical analysis was performed between focal and nonfocal patients.

*Swine based ARDS model*

Five healthy piglets (31[30-32]kg) were studied. After anesthesia induction (propofol, midazolam and sufentanyl), orotracheal intubation (diameter 8) and continuous neuromuscular blockade (cisatracurium). Animals were equipped with central venous access (internal jugular vein) and arterial femoral catheter. Electrocardiogram and arterial pressure were monitored continuously (IntelliVue MP40, Phillips and PiCCO, Pulsion SA). Pigs were placed in the supine position. Ringer Lactate (RL) was continuously infused to compensate fluid losses (5 mL.kg-1.h- 1). Hemodynamic parameters were optimized with fluid loading as required from stroke volume variation to obtain preload-independant cardiac function according to Franck-Starling concept.

Injurious mechanical ventilation was delivered with large tidal volumes (15mL.kg^-1^), zero PEEP and FiO_2_ 100% (Engström Carestation™, General Electrics Healthcare). Acid aspiration–induced ARDS was induced by intratracheal instillation of hydrochloric acid 0.05N, pH 1.41 (4ml.kg-1 bodyweight), at the level of the carina over 3 min by means of a flexible bronchoscope (Pentax). ARDS induction was considered complete when PaO_2_ to FiO_2_ ratio dropped below 100mmHg and stayed stable for at least one hour (blood samples collected at 5 cmH2O of PEEP and FiO2 of 100 %). If necessary, this operation was repeated to obtain deep hypoxemia (PaO2 to FiO2 ratio below 100 mmHg). Criteria for experimental ARDS were evaluated as recommended by the American Thoracic Society(5) at baseline in injured animals, after acid instillation in injured pigs.

Right lateral thoracic surgical drainage was operated (Seldinger Chest Drainage Kit, Portex®, Smith Medical), filled with saline 0.9% and connected to pressure transducer (Edwards Life Science). Monitored pressure was assigned to pleural pressure (Ppl). A rigid 30 cm-long catheter was inserted in the endotracheal tube (diameter 8) towards the distal lumen and connected to a second pressure transducer (Edwards Life Science). Monitored pressure was assimilated to tracheal upper airways pressure (Paw). Animals were further ventilated with VDR-4 (Volumetric Diffusive Respirator, Percussionaire® Corporation, Bird Technologies, Sandpoint, ID) with random pressure levels.

**Results**

**Table S1.** Evolution of respiratory parameters under high frequency percussive ventilation. Data are presented as median[IQR]. *Abbreviations*: PEEP: Positive end-expiratory pressure.

**Table S2.** Evolution of lung compliance and airway resistance during HFPV. **P*<0.05, Tafter *versus* T0.

**Table S3.** Evolution of alveolar recruitment and hyperinflation. Data are presented as median[IQR]. **P*<0.05 between conventional ventilation (Conv) and high frequency percussive ventilation (HFPV) end-expiratory hold (Expi) or HFPV end-inspiratory hold (Inspi).

**Table S4**. Evolution of lung volumes, masses and Hounsfield units before and after HFPV. *Abbreviations*: HU: Hounsfield unit; Mair: mass of gas; MeanCT: mean attenuation coefficient; Mtissue: mass of lung tissue; Non = non-aerated lung volume; Norm: normally-aerated lung volume; Over = overdistended lung volume; Poor = poorly-aerated lung volume; TLV: total lung volume.

**Table S5.** Correlations between hyperinflated volumes during high frequency percussive ventilation (HFPV) end-expiratory and inspiratory holds and conventional mechanical ventilation baseline volumes. *Abbreviations:* Non: non-aerated; Over: hyperinflated; Poor: poorly-aerated and Norm: normally-aerated. **P*<0.05 *versus* baseline.

**Table S6.** Correlations and Bland and Altman bias between MONITRON® monitored pressures and pleural and upper airways measured pressures during high frequency percussive ventilation (HFPV). *Abbreviations:* PEEP: positive end-expiratory pressure.

**Figure S1.** Evolution of hemodynamic parameters during experimental procedures: (A) vasopressor index; (B) vasopressor dependency index (dose-response relationship: VI over MAP ratio). **P*<0.05 *versus* T0.

**Figure S2**. Analysis of lung zones. (A) anterior; (B) middle; (C) posterior. *Abbreviations*: Vover = volume hyperinflated; Vnorm = volume normally-aerated; Vpoor = volume poorly-aerated; Vnon = volume non-aerated; conv = conventional mechanical ventilation, expi = high frequency percussive ventilation (HFPV) end-expiratory hold; inspi: HFPV end-inspiratory hold. **P*<0.05 *versus* conventional ventilation.

**Figure S3.** Evolution of lung volumes. (A) hyperinflated; (B) normally-aerated; (C) poorly-aerated; (D) non-aerated lung volumes. (E) total lung volume and (F) total gas content. *Abbreviations:* conv = conventional ventilation, expi = high frequency percussive ventilation (HFPV) end-expiratory hold; inspi: HFPV end-inspiratory hold. **P*<0.05 *versus* conventional ventilation (conv).

**Figure S4**. Correlations (A) and Bland and Altman bias (B) between minimal end-expiratory pleural pressure and HFPV PEEP considering all pairs of measurements performed during the study. (A) N = 58, red line: 95% confidence ellipsis; (B) N = 58, lines: bias (black dotted) and +2SD/-2SD limits of agreement (red dotted). *Abbreviations*: HFPV: high frequency percussive ventilation; SD: standard deviation. When considering the 58 pairs of measurements, the bias (lower to upper limits of agreement) between the absolute values of pleural minimal end-expiratory pressure and HFPV PEEP was 8.9 (-8.8 to 26.5) cmH_2_O. A good correlation was observed (*r*=0.62, *P*<10^-3^).

**Figure S5**. Correlations (A) and Bland and Altman bias (B) between maximal end-expiratory pleural pressure and HFPV PEEP considering all pairs of measurements performed during the study. (A) N = 58, red line: 95% confidence ellipsis; (B) N = 58, lines: bias (black dotted) and +2SD/-2SD limits of agreement (red dotted). *Abbreviations*: HFPV: high frequency percussive ventilation; SD: standard deviation. When considering the 58 pairs of measurements, the bias (lower to upper limits of agreement) between the absolute values of pleural maximal end-expiratory pressure and HFPV PEEP was 4.3 (-12.4 to 20.9) cmH_2_O. A good correlation was observed (*r*=0.67, *P*<10^-3^).

**Figure S6**. Correlations (A) and Bland and Altman bias (B) between minimal end-inspiratory upper airways pressure and HFPV peak pressure considering all pairs of measurements performed during the study. (A) N = 54, red line: 95% confidence ellipsis; (B) N = 54, lines: bias (black dotted) and +2SD/-2SD limits of agreement (red dotted). *Abbreviations*: HFPV: high frequency percussive ventilation; SD: standard deviation. When considering the 54 pairs of measurements, the bias (lower to upper limits of agreement) between the absolute values of upper airways minimal end-inspiratory pressure and HFPV peak pressure was 9.3 (2.7 to 15.8) cmH_2_O. A good correlation was observed (*r*=0.73, *P*<10^-3^).

**Figure S7**. Correlations (A) and Bland and Altman bias (B) between maximal end-inspiratory upper airways pressure and HFPV peak pressure considering all pairs of measurements performed during the study. (A) N = 55, red line: 95% confidence ellipsis; (B) N = 55, lines: bias (black dotted) and +2SD/-2SD limits of agreement (red dotted). *Abbreviations*: HFPV: high frequency percussive ventilation; SD: standard deviation. When considering the 54 pairs of measurements, the bias (lower to upper limits of agreement) between the absolute values of upper airways maximal end-inspiratory pressure and HFPV peak pressure was 3.8 (-4.5 to 12.0) cmH_2_O. A good correlation was observed (*r*=0.71, *P*<10^-3^).

**Figure S8**. Correlations (A) and Bland and Altman bias (B) between minimal end-inspiratory pleural pressure and HFPV mean pressure considering all pairs of measurements performed during the study. (A) N = 58, red line: 95% confidence ellipsis; (B) N = 58, lines: bias (black dotted) and +2SD/-2SD limits of agreement (red dotted). *Abbreviations*: HFPV: high frequency percussive ventilation; SD: standard deviation. When considering the 58 pairs of measurements, the bias (lower to upper limits of agreement) between the absolute values of pleural minimal end-inspiratory pressure and HFPV mean pressure was 12.2 (2.2 to 22.2) cmH_2_O. A weak correlation was observed (*r*=0.49, *P*<10^-3^).

**Figure S9**. Correlations (A) and Bland and Altman bias (B) between minimal end-expiratory upper airways pressure and HFPV PEEP considering all pairs of measurements performed during the study. (A) N = 50, red line: 95% confidence ellipsis; (B) N = 50, lines: bias (black dotted) and +2SD/-2SD limits of agreement (red dotted). *Abbreviations*: HFPV: high frequency percussive ventilation; SD: standard deviation. When considering the 50 pairs of measurements, the bias (lower to upper limits of agreement) between the absolute values of upper airways minimal end-expiratory pressure and HFPV PEEP was 4.0 (-10.2 to 18.1) cmH_2_O. A good correlation was observed (*r*=0.64, *P*<10^-3^).

**Figure S10**. Correlations (A) and Bland and Altman bias (B) between maxmal end-expiratory upper airways pressure and HFPV PEEP considering all pairs of measurements performed during the study. (A) N = 50, red line: 95% confidence ellipsis; (B) N = 50, lines: bias (black dotted) and +2SD/-2SD limits of agreement (red dotted). *Abbreviations*: HFPV: high frequency percussive ventilation; SD: standard deviation. When considering the 50 pairs of measurements, the bias (lower to upper limits of agreement) between the absolute values of upper airways maximal end-expiratory pressure and HFPV PEEP was -6.2 (-13.8 to 1.4) cmH_2_O. A good correlation was observed (*r*=0.64, *P*<10^-3^).

**Figure S11**. Volume distribution of CT attenuations (Hounsfield units (HU)) under conventional ventilation (Conv) and high frequency percussive ventilation (HFPV; Expi: HFPV end-expiratory hold; Inspi: HFPV end-inspiratory hold) in patients with nonfocal ARDS. Lung aeration was reported as hyperinflated (-1000 to -900 HU); normally-aerated (Norm, -900 to -500 HU); poorly-aerated (Poor, -500 to -100 HU) and non-aerated (Non, -100 to +100 HU).

**Table S1**

|  | T10 | T20 | T30 | T40 | T50 | T60 | *P* |
| --- | --- | --- | --- | --- | --- | --- | --- |
| Percussive Frequency | 590[563-663] | 590[560-658] | 600[565-648] | 575[565-640] | 575[568-630] | 580[548-655] | 0.92 |
| Convective Frequency | 15[11-15] | 14[11-16] | 15[13-18] | 15[12-18] | 14[12-17] | 14[13-16] | 0.97 |
| PEEP, cmH_2_O | 23[19-28] | 23[21-27] | 22[22-29] | 23[22-29] | 23[21-27] | 27[22-28] | 0.98 |
| Mean Pressure, cmH_2_O | 31[23-33] | 31[27-33] | 30[30-33] | 30[30-31] | 30[29-32] | 31[30-32] | 0.93 |
| Peak Pressure, cmH_2_O | 63[57-68] | 65[61-69] | 61[53-67] | 62[58-65] | 61[58-67] | 60[58-67] | 0.98 |
| Expiratory Time, s | 2.1[1.4-3.1] | 2.2[1.3-3.8] | 1.6[1.4-2.4] | 1.6[1.4-2.8] | 2.1[1.6-2.8] | 2.0[1.5-2.8] | 0.99 |
| Inspiratory Time, s | 2.3[1.7-2.7] | 2.0[1.4-2.8] | 1.9[1.8-2.3] | 2.0[2.0-2.4] | 2.1[1.8-2.4] | 2.2[2.0-2.7] | 0.98 |

**Table S2**

| *Compliance* (mL.cmH_2_O^-1^) | |  | | *Resistance* (cmH_2_O.mL^-1^.s^-1^) | | |
| --- | --- | --- | --- | --- | --- | --- |
| T0 | Tafter | |  | | T0 | Tafter |
| 20.5[17.8-28.8] | 28.5[24.5-32.3]* | |  | | 14.5[11.0-15.5] | 13.5[12.5-14.0] |

**Table S3**

|  | Expi *versus* Conv |  | Inspi *versus* Conv | |  |
| --- | --- | --- | --- | --- | --- |
|  |  |  |  | |  |
| *Recruitment* |  |  |  | |  |
| **Changes in non-aerated lung volumes** | | | | | |
| Volume, mL | 394[257-568]* |  | 402[312-537]* | |  |
| Percentage, % | 12.0[8.5-18.0]* |  | 12.5[9.3-16.8]* | |  |
| **Changes in non- and poorly-aerated lung volumes** | | | | | |
| Volume, mL | 358[86-450] |  | 385[63-472] | |  |
| Percentage, % | 12.0[5.0-18.0] |  | 11.0[6.0-22.0] | |  |
| *Hyperinflation* |  |  | |  | |
| Volume, mL | 38[0-82] |  | 112[70-164] | |  |
| Percentage, % | 2.0[0.5-2.5] |  | 3.0[2.5-4.0] | |  |

**Table S4**

|  |  | Conventional  End Expiratory | HFPV  End Expiratory | HFPV  End Inspiratory |  |
| --- | --- | --- | --- | --- | --- |
| TLV, mL |  | 3169[2612-4802] | 3804[2568-4531] | 4186[2770-4872] |  |
| Gas volume, mL |  | 1441[909-2328] | 1844[1138-2631] | 2501[1317-3010] |  |
| Tissue volume, mL |  | 1959[1635-2230] | 1677[1362-2083] | 1714[1296-2083] |  |
| Mair, g |  | 1315[1151-1453] | 1358[1108-1630] | 1395[1149-1564] |  |
| Mtissue, g |  | 579[471-775] | 313[206-542] | 313[191-428] |  |
| MeanCT, HU |  | -444[-521- -341] | -474[-534- -447] | -532[-605- -502] |  |
| Over, mL |  | 199[78-351] | 111[103-366] | 305[180-490] |  |
| Norm, mL |  | 1473[893-2208] | 1903[1100-2442] | 2439[1350-2748] |  |
| Poor, mL |  | 763[733-876] | 821[752-1292] | 780[693-1214] |  |
| Non, mL |  | 843[710-1183] | 511[356-794] | 490[324-627] |  |

**Table S5**

|  | HFPV end-expiratory hold | |  | HFPV end-inspiratory hold | |
| --- | --- | --- | --- | --- | --- |
|  | *r* | *P* |  | *r* | *P* |
| Non | 0.536 | 0.24 |  | 0.238 | 0.58 |
| Poor | -0.126 | 0.80 |  | -0.383 | 0.35 |
| Norm | 0.786 | 0.05* |  | 0.714 | 0.06 |
| Over | 0.786 | 0.05* |  | 0.880 | 0.01* |

**Table S6**

| *A. Pleural Pressures* | | | | |
| --- | --- | --- | --- | --- |
|  | **MONITRON ®** | | | |
|  | PEEP | | Mean Pressure | |
|  | **Pleural Pressures** | | | |
|  | End-Expiratory Min | End-Expiratory Max | End-Inspiratory Min | End-Inspiratory Max |
| ***Number of pairs*** | 58 | 58 | 58 | 58 |
| ***Spearman’s Correlation*** | | | | |
| *r* | 0.62 | 0.67 | 0.49 | 0.19 |
| *P* | <10^-3^ | <10^-3^ | <10^-3^ | 0.16 |
| ***Bland and Altman*** | | | | |
| Bias | 8.9 | 4.3 | 12.2 | 6.1 |
| 95% Limits of Agreement | -8.8 to 26.5 | -12.4 to 20.9 | 2.2 to 22.2 | -7.3 to 19.5 |

| *B. Upper Airways Pressures* | | | | |
| --- | --- | --- | --- | --- |
|  | **MONITRON ®** | | | |
|  | PEEP | | Peak Pressure | |
|  | **Upper Airways Pressures** | | | |
|  | End-Expiratory Min | End-Expiratory Max | End-Inspiratory Min | End-Inspiratory Max |
| ***Number of pairs*** | 50 | 50 | 54 | 55 |
| ***Spearman’s Correlation*** | | | | |
| *r* | 0.64 | 1 | 0.73 | 0.71 |
| *P* | <10^-3^ | <10^-3^ | <10^-3^ | <10^-3^ |
| ***Bland and Altman*** | | | | |
| Bias | 4.0 | -6.2 | 9.3 | 3.8 |
| 95% Limits of Agreement | -10.2 to 18.1 | -13.8 to 1.4 | 2.7 to 15.8 | -4.5 to 12.0 |

**Figure S1**

**Figure S2**

**Figure S3**

**Figure S4**

**
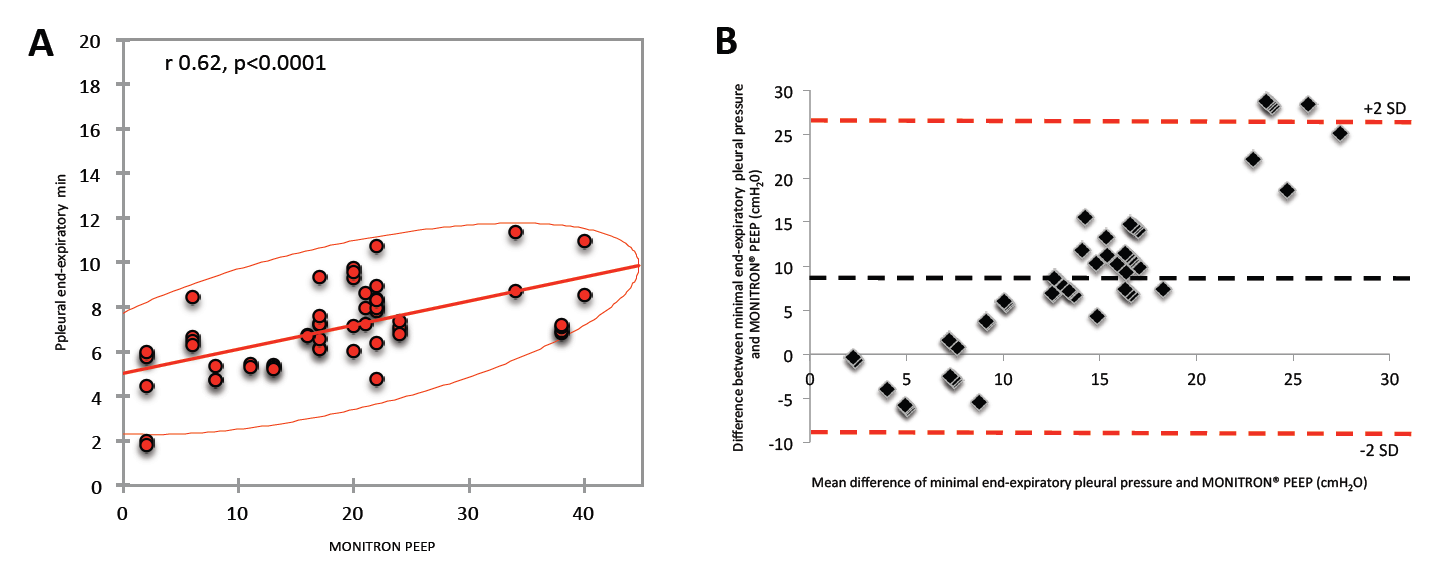
Figure S5**

**
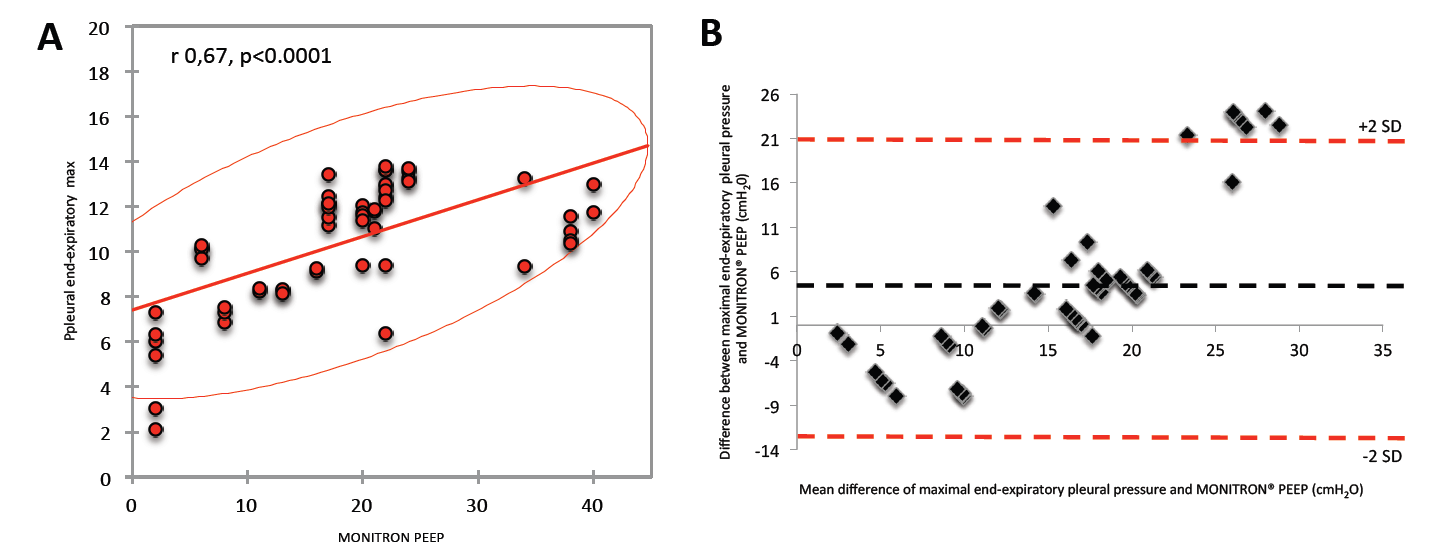
**

**Figure S6**

**
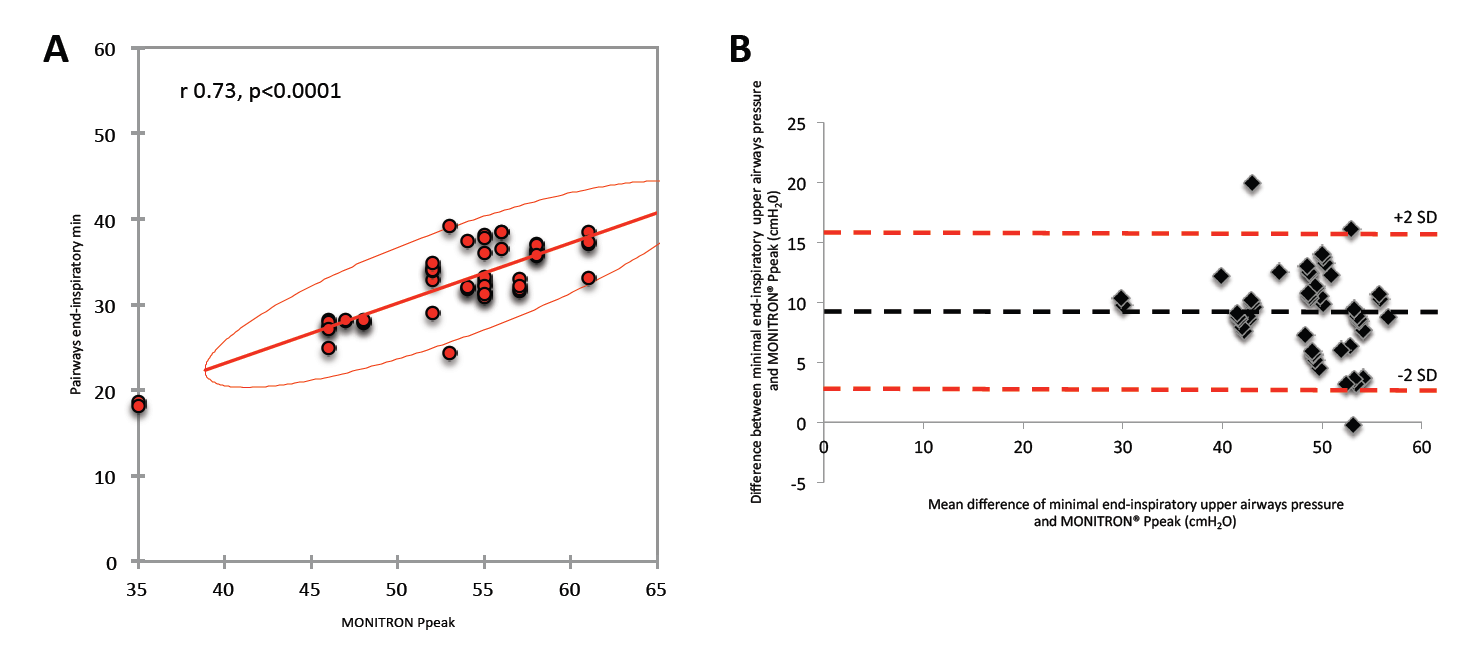
**

**Figure S7**

**
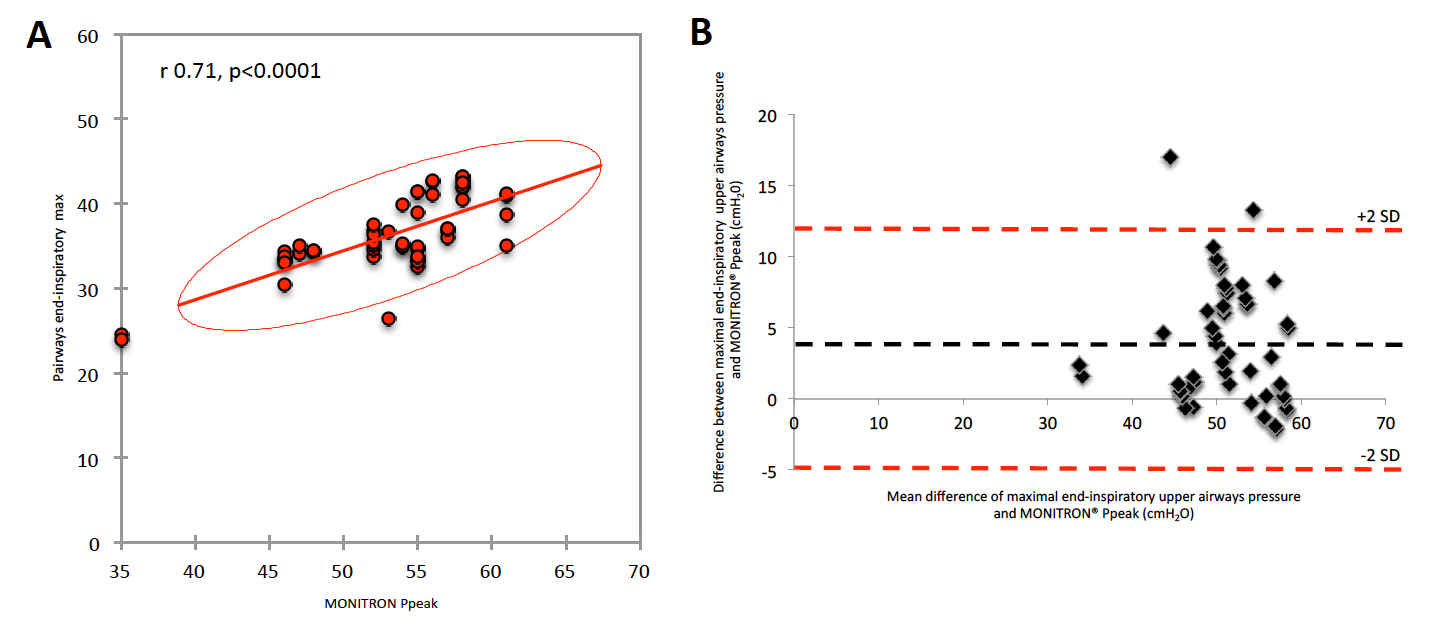
**

**Figure S8**

**
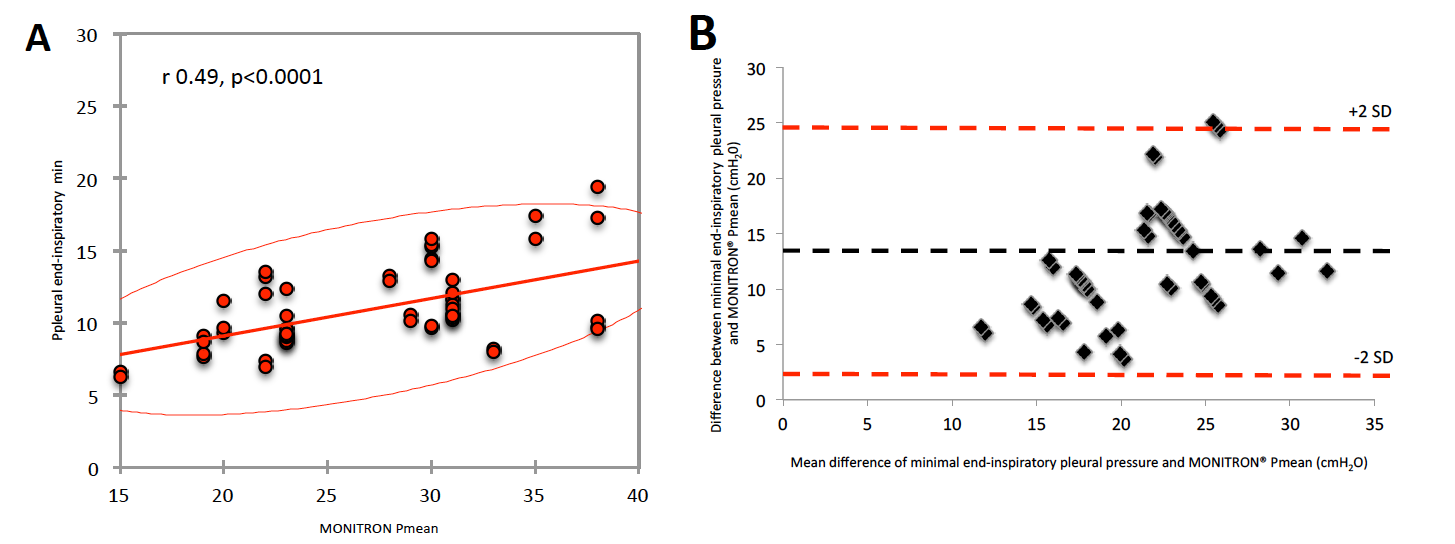
**

**Figure S9**

**
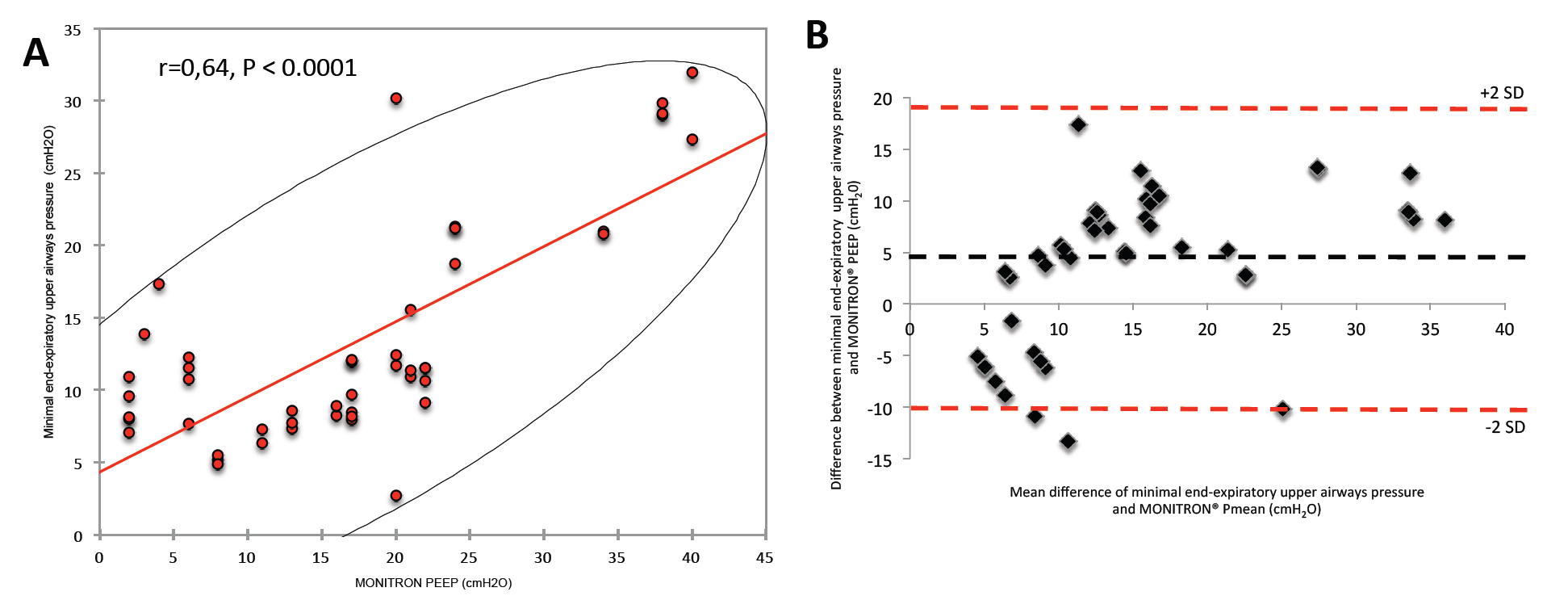
**

**Figure S10**

**
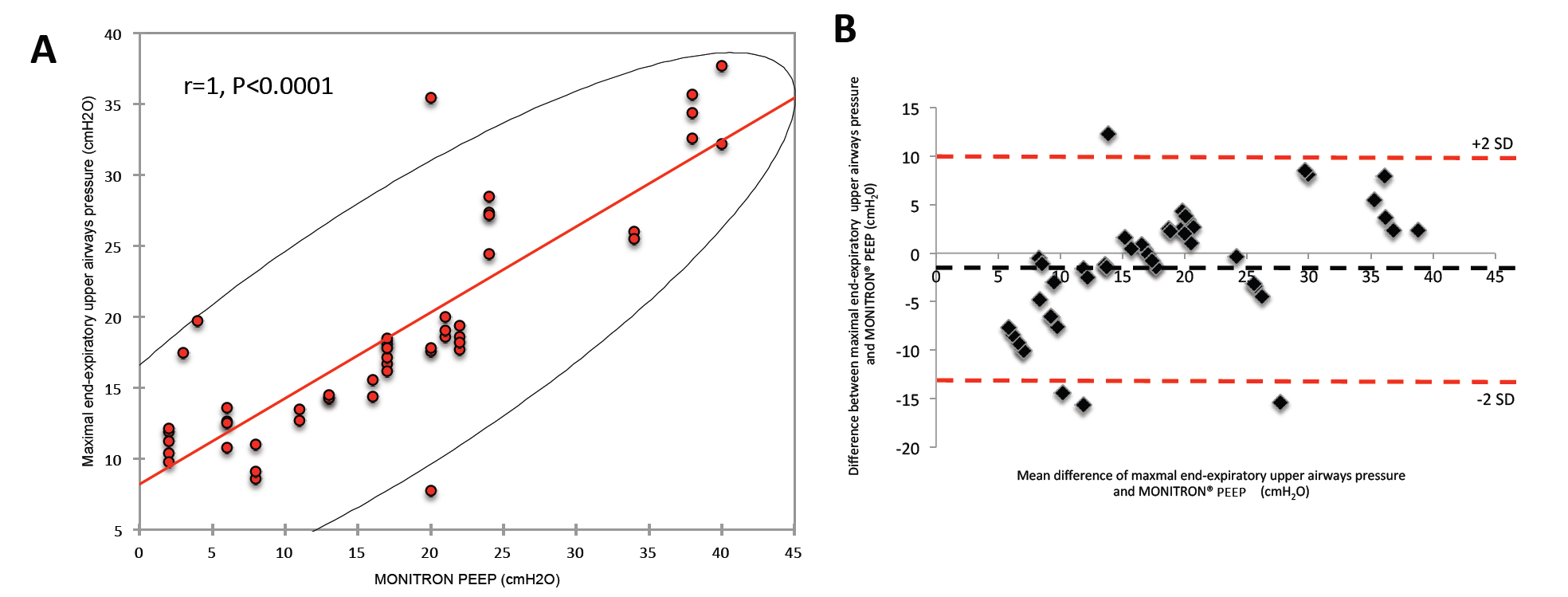
**

**Figure S11**


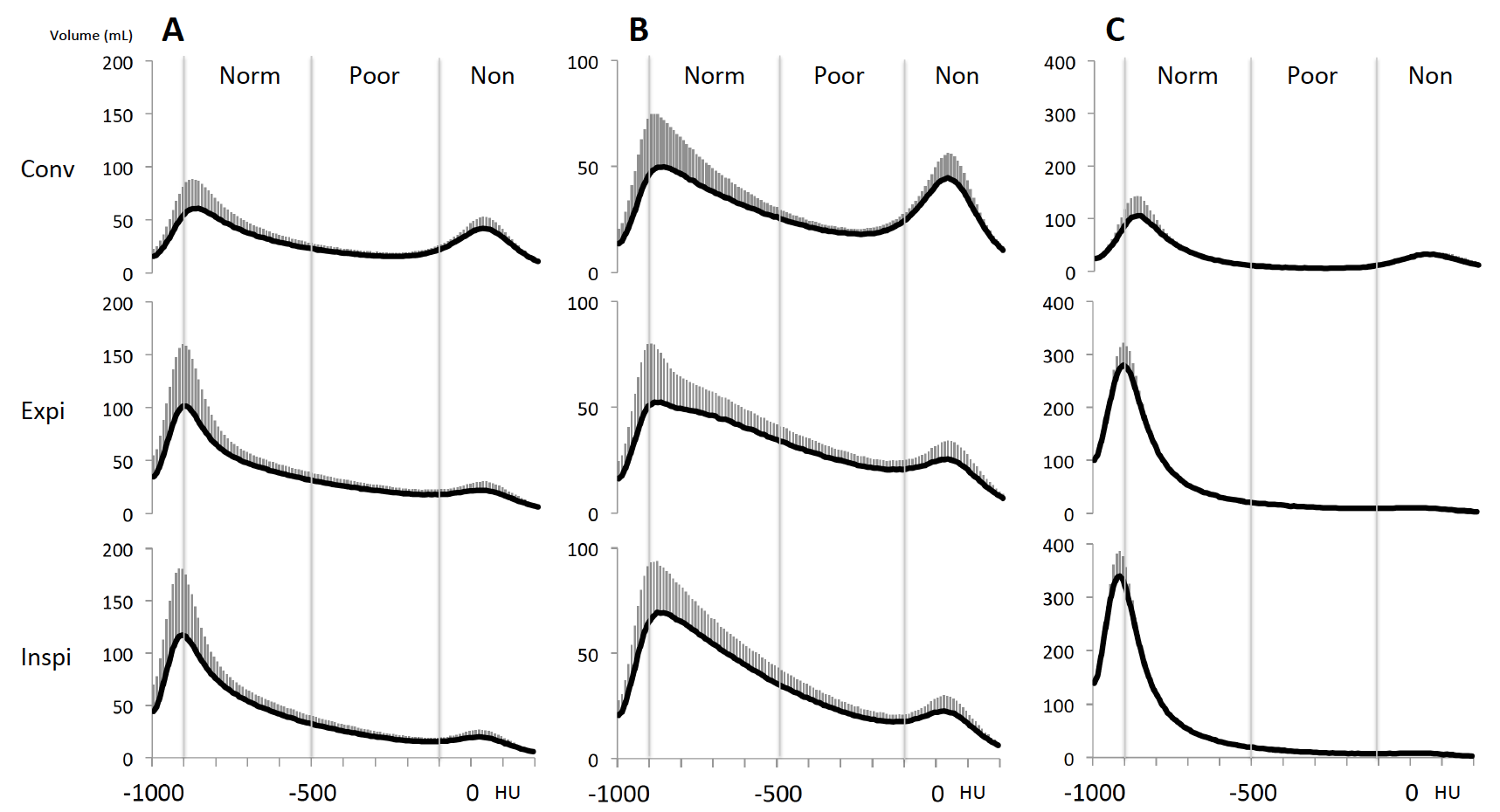

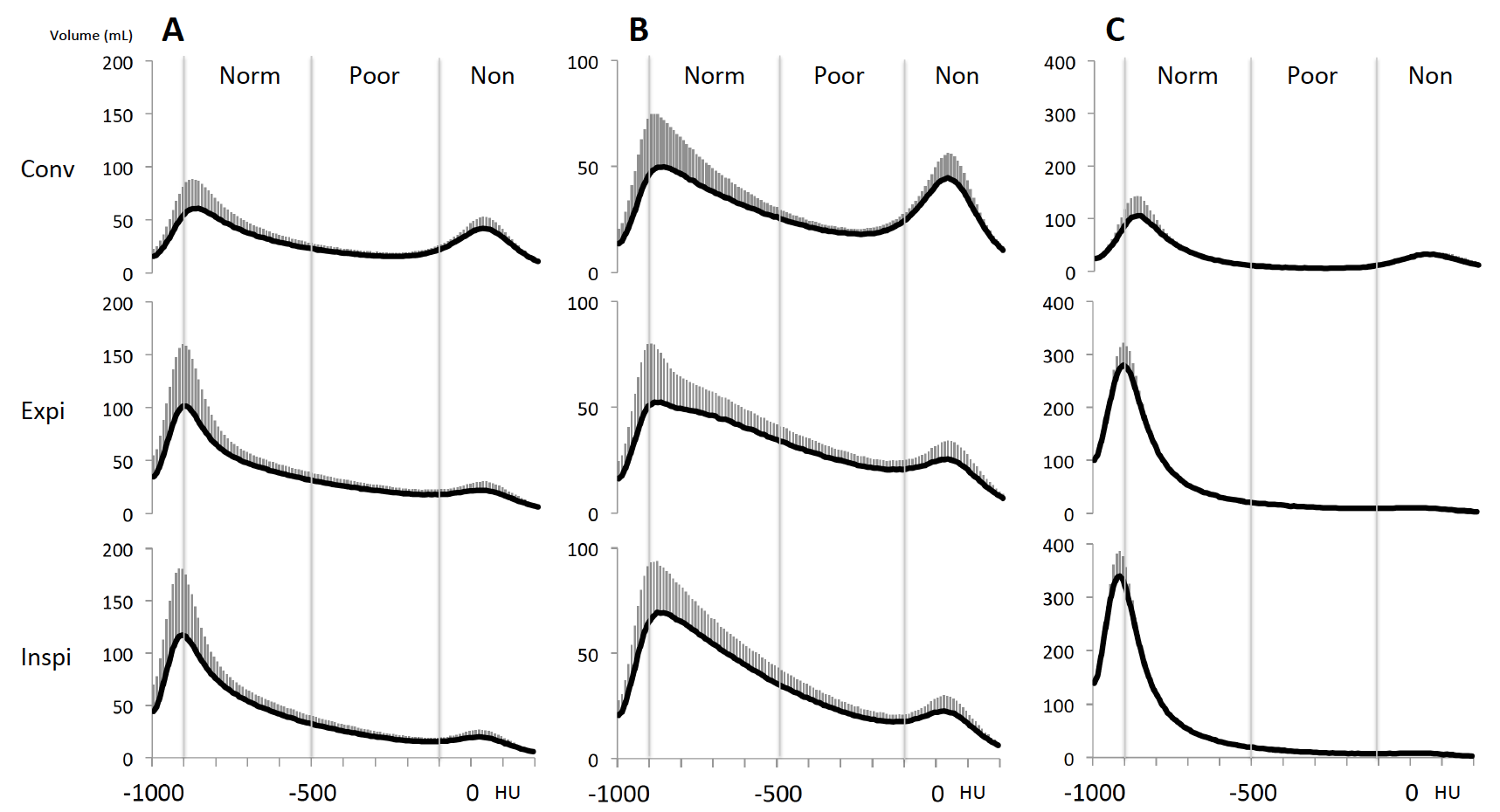


1. Force ADT, Ranieri VM, Rubenfeld GD, Thompson BT, Ferguson ND, Caldwell E, Fan E, Camporota L, Slutsky AS. Acute respiratory distress syndrome: the Berlin Definition. *JAMA : the journal of the American Medical Association* 2012; 307: 2526-2533.

2. Mercat A, Richard JC, Vielle B, Jaber S, Osman D, Diehl JL, Lefrant JY, Prat G, Richecoeur J, Nieszkowska A, Gervais C, Baudot J, Bouadma L, Brochard L, Expiratory Pressure Study G. Positive end-expiratory pressure setting in adults with acute lung injury and acute respiratory distress syndrome: a randomized controlled trial. *JAMA : the journal of the American Medical Association* 2008; 299: 646-655.

3. Constantin JM, Grasso S, Chanques G, Aufort S, Futier E, Sebbane M, Jung B, Gallix B, Bazin JE, Rouby JJ, Jaber S. Lung morphology predicts response to recruitment maneuver in patients with acute respiratory distress syndrome. *Critical care medicine* 2010; 38: 1108-1117.

4. Cruz DN, Antonelli M, Fumagalli R, Foltran F, Brienza N, Donati A, Malcangi V, Petrini F, Volta G, Bobbio Pallavicini FM, Rottoli F, Giunta F, Ronco C. Early use of polymyxin B hemoperfusion in abdominal septic shock: the EUPHAS randomized controlled trial. *JAMA : the journal of the American Medical Association* 2009; 301: 2445-2452.

5. Matute-Bello G, Downey G, Moore BB, Groshong SD, Matthay MA, Slutsky AS, Kuebler WM, Acute Lung Injury in Animals Study G. An official American Thoracic Society workshop report: features and measurements of experimental acute lung injury in animals. *American journal of respiratory cell and molecular biology* 2011; 44: 725-738.
